# An ALBI- and Ascites-Based Model to Predict Survival for BCLC Stage B Hepatocellular Carcinoma

**DOI:** 10.1155/2022/1801230

**Published:** 2022-07-07

**Authors:** Cheng He, Jing Yang, Zheng Jin, Ying Zhu, Wei Hu, Lingfeng Zeng, Xiaocheng Li

**Affiliations:** ^1^Department of Hepatobiliary Surgery, Affiliated Hospital of Xiangnan University, Chenzhou, Hunan, China; ^2^Department of Intensive Care Unit, Affiliated Hangzhou First People's Hospital, Zhejiang University School of Medicine, Hangzhou, Zhejiang, China; ^3^Department of Gastroenterology, Affiliated Hangzhou First People's Hospital, Zhejiang University School of Medicine, Hangzhou, Zhejiang, China; ^4^Li Ka Shing Institute of Health Sciences (LiHS), Faculty of Medicine, The Chinese University of Hong Kong, Shatin, Hong Kong, China; ^5^Department of Hepatobiliary Surgery, The First Affiliated Hospital of Hunan University of Medicine, Huaihua, Hunan, China

## Abstract

**Background:**

We aimed to develop a predictive model constituted with the ALBI grade, the ascites, and tumor burden related parameters in patients with BCLC stage B HCC.

**Methods:**

Patients diagnosed as the BCLC stage B HCC were collected from a retrospective database. Construction and validation of the predictive model were performed based on multivariate Cox regression analysis. Predictive accuracy, discrimination (c-index), and fitness performance (calibration curve) of the model were compared with the other eight models. The decision curve analysis (DCA) was used to evaluate the clinical utility.

**Results:**

A total of 1773 patients diagnosed as BCLC stage B HCC between 2007 and 2016 were included in the present study. The ALBI-AS grade, the AFP level, and the 8-and-14 grade were used for the development of a prognostic prediction model after multivariate analysis. The area under the receiver operator characteristic curve (AUROC) for overall survival at 1, 2, and 3 years predicted by the present model were 0.73, 0.69, and 0.67 in the training cohort. The concordance index (c-index) and the Aiken information criterion (AIC) were 0.68 and 6216.3, respectively. In the internal and external validation cohorts, the present model still revealed excellent predictive accuracy, discrimination, and fitness performance. Then the ALBI-AS based model was evaluated to be superior to other prognostic models with the highest AUROC, c-index, and lowest AIC values. Moreover, DCA also demonstrated that the present model was clinically beneficial.

**Conclusion:**

The ALBI-AS grade is a novel predictor of survival for patients with BCLC stage B HCC.

## 1. Introduction

Hepatocellular carcinoma (HCC) is a fetal disease worldwide with leading mortality [1, 2]. The prognosis of HCC remains poor due to the relatively high proportion of unresectable disease at the time of diagnosis. The Barcelona clinic liver cancer (BCLC) staging system has been largely used in clinical practice [3]. Patients of BCLC stage B are considered unsuitable for curative treatment and their overall survival rate is highly variable [4]. The wide variations in overall survival are mainly due to the heterogeneity of liver function and tumor burdens. Therefore, several subclassification systems or risk predication models for BCLC stage B HCC patients were proposed based on the parameters related to liver function and tumor burden [5–9].

The Child–Pugh (CP) grade is the most widely used tool for the assessment of liver function, and it has been applied in several prognosis prediction models for BCLC stage B HCC patients, such as the BCLC stage B subclassification system and the SNACOR model [4, 7]. However, there are several limitations to the application of CP grade in HCC patients. The CP grade has five parameters, the bilirubin, albumin, prothrombin time, hepatic encephalopathy, and ascites. The selection of cut points for the continuous variables (the bilirubin, albumin, and prothrombin time), and the subjectivity in the use of the categorical variables (hepatic encephalopathy and ascites), leads to decreased discrimination power for the prognosis prediction [10]. Recently, the Albumin-Bilirubin (ALBI) grade was reported to be a simple method for evaluating liver function and prognosis in HCC patients. The ALBI grade only contains two objective parameters of the Child–Pugh grade, and that was evaluated to have better performance in terms of prognosis prediction compared with the Child–Pugh grade [11]. The ALBI grade has been incorporated into the prognostic models for BCLC stage B HCC patients or patients who underwent TACE.

The ascites variable is eliminated in the ALBI grade. The reasons for that are as follows: the grading of ascites was believed to be highly subjective; the distinction between mild and moderate ascites was subject to interobserver variability; and the ascites and serum albumin level were interrelated. Actually, the proportion of patients with moderate to large amounts of ascites is low in the BLCL stage B HCC population. Therefore, the ascites variable could be set as a binary variable (with or without ascites), which reduced the subjectivity of judging the amount of ascites [10]. The production of ascites and its volume mainly depend on the portal vein pressure, though that might be influenced by the albumin level [12]. And the ascites always predicted the prognosis more accurately than the albumin level in previous HCC-related risk models. Additionally, ascites has been incorporated into several HCC-related prognostic models (such as HCC with portal vein tumor thrombus, HCC after ablation, HCC after palliative treatments, and BCLC stage C HCC) [13–17]. Therefore, it appears arbitrary and crude to eliminate the ascites variable from the risk prediction model for BCLC stage B HCC patients. The sum of the size of the largest tumor and the number of tumors was always used for the evaluation of the tumor burden. For instance, the up-to-seven criteria patients underwent liver transplantation and the up-to-eleven criteria patients underwent TACE [8, 18]. However, the optimal cutoff point of that parameter in the BCLC stage B HCC patients are still on debate. In the present study, the ALBI, the ascites, the size of the tumor, the number of tumors and other clinical parameters were all used for the development of a prognostic model for the BCLC stage B HCC patients.

## 2. Methods

### 2.1. Study Population

Between January 2007 and December 2016, 2020 consecutive patients with BCLC Stage B HCC were collected from a retrospective database [19]. As described in the databases and the previous studies, 1606 patients from the Sun Yat-sen University Cancer Center were used for the development of training and internal validation cohort. The remaining 414 patients from other hospitals were utilized for the external validation.

The inclusion criteria were set as: (a) HCC diagnosed as the AASLD guidelines; (b) Child–Pugh grade A or B and ECOG performance status of 0; (c) patients no less than 18 years old; (d) multiple tumors without blood vessels or lymphatic/extrahepatic metastasis. The exclusion criteria were: (a) patients with a history of malignant tumors other than HCC; (b) recurrent liver cancer or liver cancer with vascular invasion or lymphatic/extrahepatic metastasis; (c) Child–Pugh grade C; (d) patients with hepatic encephalopathy/refractory ascites/gastrointestinal hemorrhage; (e) patients with immunodeficiency or autoimmune diseases.

### 2.2. Development of the Prognostic Model

The demographics and biochemistry tests of patients were extracted for analysis. The ALBI score was calculated using the following formula: linear predictor = (log_10_ bilirubin × 0.66) + (albumin × −0.085), where bilirubin is in (mol/L) and albumin in (g/L) [10]. We redefined the cutoff value of the ALBI score for grading by the X-tile. The AST to platelet ratio index (APRI) was calculated as the formula: ((AST/upper limit of normal)/platelet count (10^9^/L)) × 100 [20]. The Child–Pugh grade was evaluated by the laboratory data of AST, albumin, and total bilirubin, and clinical data of hepatic encephalopathy and ascites. The ascites was defined as the radiological ascites. The 8-and-14 grade was evaluated by the sum of the size of the largest tumor and the number of tumors. The cutoff value was defined by the X-tile. Overall survival was the primary outcome, and that was defined as the time span from the HCC diagnosis to the last follow-up. The prognostic value of the above laboratory and clinical variables was evaluated, respectively. The independent prognostic variables would be put into the model. And the combination variables, not separately, would be used for the development of the model and the ALBI grade, instead of the albumin and total bilirubin, would be put into the model if it fulfilled the criteria.

### 2.3. Statistical Analysis

The continuous variables were presented as the mean with standard deviation or median with interquartile range (IQR). The categorical variables were presented as the number (percent). We used the Kaplan–Meier method to create the cumulative survival curve. Then the survival rate of patients was compared by the method of the log-rank test. The Cox regression analyses were used to evaluate the prognostic value of the clinical factors for the development of the model. In the stepwise backward selection manner, the multivariable analyses identified the independent prognostic factors from the variables that achieved statistical significance (*p* < 0.05) in the univariable analyses. A nomogram was generated by the Cox regression coefficients. The discrimination and fitness performance of the prognostic model were evaluated by the concordance index (c-index) and the Aiken information criterion (AIC) separately. And the accuracy for the outcome prediction was evaluated by the area under the receiver operator characteristic curve (AUROC). We compared the present model with other models such as the HAP score, the mHAP II score, the ALBI-TAE model, the up-to-seven system, the four-and-seven system, the six-and-twelve score system, the BCLC-B substaging system, and the new BCLC-B substaging system [7–9, 18, 21–24]. Then the clinical utilities of the present model were evaluated by the decision curve analysis (DCA). The statistical analyses were done by using R (version 3.5). Statistical significance was set at *p* < 0.05.

## 3. Results

### 3.1. Patients

After the patient's selection, a total of 1773 patients fulfilled the inclusion criteria. There were 903 patients that formed the training cohort, and 527 patients were used for internal validation and 343 patients for external validation. The baseline characteristics of the patients from the training cohort were presented in Table 1, and the baseline characteristics of the internal and external validation cohorts were shown in the Supplementary Table 1. Most of the patients from the training cohort were male (90.9%), and most of them were HBV infected (87.7%). The median follow-up period was 16.6 months in the training cohort, 17.0 months in the internal validation cohorts, and 17.5 months in the external validation cohort. More than 80% of patients from the training cohort were Child–Pugh grade B. However, the majority of patients from the internal and external validation cohorts were Child–Pugh grade A. More than 60% of patients had at least 3 lesions in the whole cohort. The median size of tumors ranged between 63 and 67 mm in training, internal, and external cohorts, and there were 3% to 5% of the patients with ascites. The mean ALBI score was −2.4 in the training cohort, −2.5 in the internal validation cohort, and −2.4 in the external validation cohort.

### 3.2. Survival Analyses and Development of the Prognostic Model

**Figure 1:**
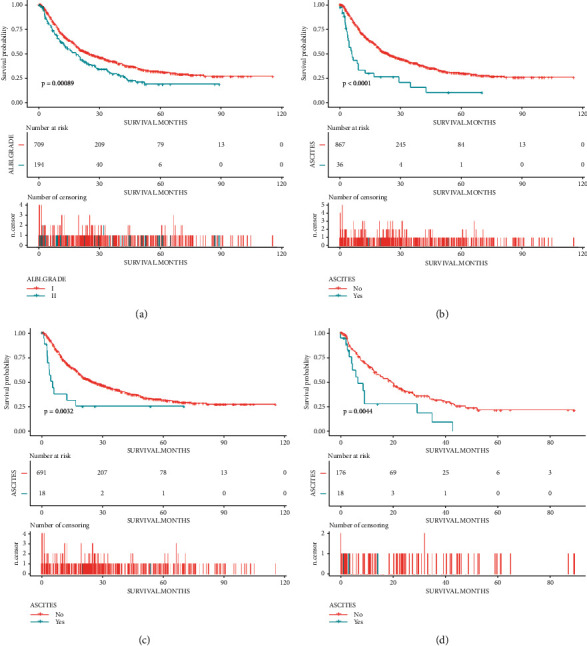
Kaplan–Meier curves of overall survival in patients with BCLC stage B HCC stratified by (a) the ALBI grade and (b) the ascites. And the Kaplan–Meier curves of overall survival in patients of (c) ALBI grade I subgroup and (d) ALBI grade II subgroup, stratified by the ascites.

The cut point for the ALBI score in the study of Johnson et al. was set as −2.60 and −1.39 (less than −2.60 for grade 1, −2.60 to −1.39 for grade 2, and more than −1.39 for grade 3), and their study was based on the analysis for all stages of HCC [10]. The study of Lee et al. for BCLC stage B HCC, which used the similar grading method for ALBI as the study of Johnson et al., revealed that there was no significant difference in terms of survival between the patients of ALBI stage 2 and 3 [9]. They combined the patients of ALBI grades 2 and 3 into one group for the analysis. The present study also focused on the BCLC stage B HCC; therefore, we divided the patients into two groups according to the ALBI score, and we defined the cutoff point by the use of the X-tile. As presented in Supplementary Figure 1, the cutoff point in the internal cohort (the training and internal validation cohorts) and external cohort was all defined as −2.3. The ALBI score of less than −2.3 was defined as the ALBI grade I, and the other patients were defined as the grade II. As shown in Figure 1(a), there was a significant difference in terms of overall survival between the ALBI grade I and II groups (*p* < 0.001).

**Figure 2:**
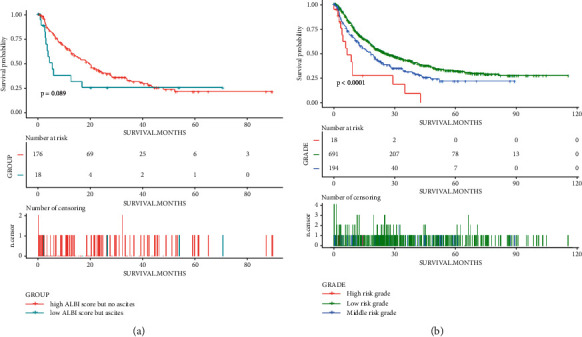
(a) Kaplan–Meier curve of overall survival in patients from the group of high ALBI score and no ascites and group of low ALBI score and ascites. Kaplan–Meier curve of overall survival in patients from the low-risk (low ALBI score and no ascites), high risk (high ALBI score and ascites) and the rest patients (the middle risk).

Then we compared the survival time between the patients with and without ascites, as shown in Figure 1(b), patients without ascites had a significantly better overall survival than those with ascites (*p* < 0.001). And the prognostic value of ascites was confirmed in the analyses for the subgroup of ALBI grade I or II patients (Figures 1(c) and 1(d)). Therefore, we used the ALBI grade and the information of ascites for the assessment of the liver function. Next, we explored the method for the combination of the ALBI grade and the ascites. We found out that patients with a low ALBI score and no ascites had the best prognosis (those patients would be defined as the low-risk grade), and patients with a high ALBI score and ascites had the worst prognosis (high risk grade). There was no significant difference in terms of the overall survival of patients with high ALBI score but no ascites and low ALBI score but ascites, therefore we defined those patients as the middle grade (Figure 2).

**Figure 3:**
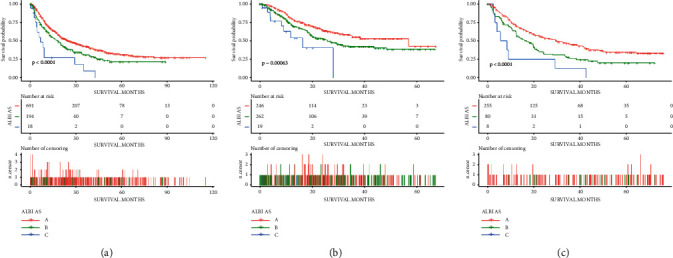
Kaplan–Meier curves of overall survival in patients stratified by the ALBI-AS grade in the (a) training, (b) internal validation, and (c) external validation cohorts.

As presented in Table 2, we combined the ALBI grade and the ascites into a new variable, the ALBI-AS grade (where grade A represents the low-risk grade, grade B for the middle risk, and grade C for the high risk). The ALBI-AS grade was simpler than the Child–Pugh grade and a little more complicated than the ALBI grade, but more comprehensive and accurate, in terms of assessment for the liver function.  Figure 3 shows the prognostic value of the ALBI-AS grade in the training, internal validation, and external validation cohorts. Observed survival rates at 1 and 3 years were 69.1% and 42.9% for the ALBI grade I patients, 61.2% and 29.4% for the ALBI grade II patients. And the observed survival rates at 1 and 3 years were 69.9% and 42.4% for the ALBI-AS grade A patients, respectively, and 61.4% and 9.1% for the ALBI-AS grade C patients. Similar to the up-to-seven criteria or up-to-eleven criteria in the previous studies, the sum of the size of the largest tumor and the number of tumors was used for the assessment of the tumor characteristics. With 8 and 14 as the cutoff points defined by the X-tile (the Supplementary Figure 2), we used the 8-and-14 grade for the development of the predictive model.

**Figure 4:**
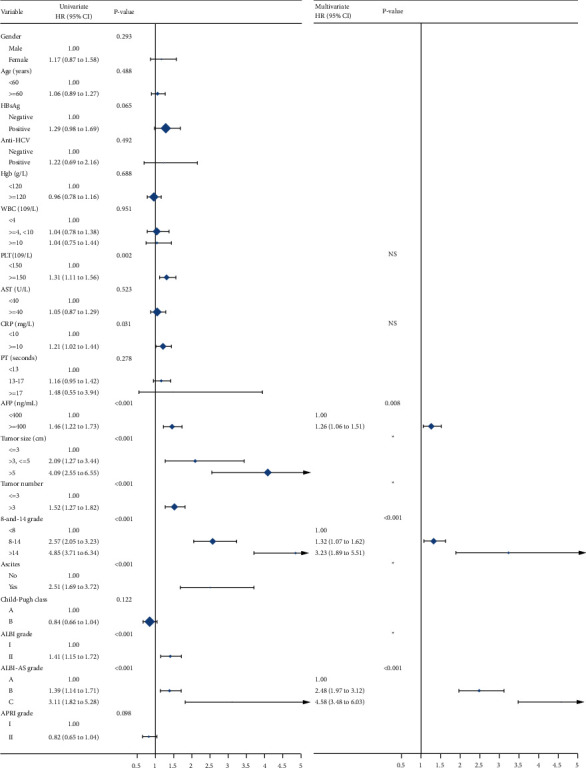
The univariate (left panel) and multivariable (right panel) survival analyses. ^*∗*^ represents significance in the univariate analyses but not included in the multivariable analyses.

As shown in Figure 4, the univariate analysis revealed that nine variables including the baseline serum PLT level, the baseline CRP level, the baseline AFP level, the tumor size, the tumor number, the 8-and-14 grade, ascites, the ALBI grade, and the ALBI-AS grades were evaluated to be associated with overall survival. Then the baseline PLT, CRP, AFP level, and the 8-and-14 grade, the ALBI-AS grade were put into the multivariable analyses. After the multivariable Cox survival analyses, the AFP level, the 8-and-14 grade, and the ALBI-AS grade were finally selected for the development of the model. The Supplementary Figure 3 showed the prognostic value of the AFP level, the 8-and-14 grade, and the ALBI-AS grade separable.

**Figure 5:**
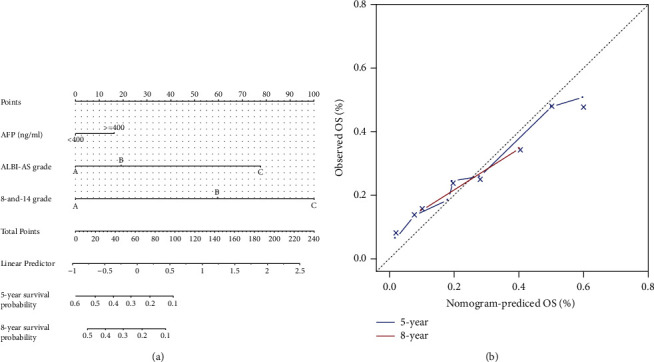
Nomogram (left panel) to predict 5-year and 8-year overall survival. Calibration plot (right panel) at 5 and 8 years for the final model.

Then we formulated a nomogram with the three selected prognostic factors, as shown in Figure 5. The associated c-index was 0.68 (95% confidence interval (CI), 0.66–0.70), which showed the nomogram model could predict 68% of the individual death probability. The calibration curves showed a high consistency in the prediction of the 5-and 8-year overall survival.

### 3.3. Validation of the Model and Comparison with Other Models

**Figure 6:**
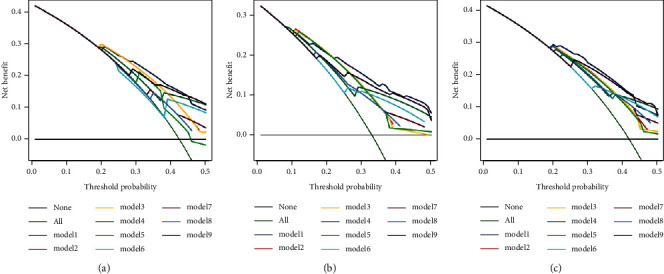
The decision analysis curve in the (a) training, (b) internal validation, and (c) external validation cohorts. Model 1: the present model; model 2: up-to-seven; model 3: four-and-seven; model 4: six-and-twelve; model 5: BCLC-B substaging system; model 6: new BCLC-B substaging system; model 7: HAP; model 8: mHAP II; model 9: ALBI-TAE model.

We validated the efficacy of the present model in the internal and external validation cohorts. As shown in Table 3, the c-index and AIC in the present model were 0.68 and 6216.3. And the 1- to-3-year AUROC ranged from 0.67 to 0.73. The c-index and AIC in the internal validation cohort were 0.70 and 2306.2, and those in the external validation cohort were 0.67 and 2056.6. And the AUROC of three years in the internal and external validation cohort presented a relatively high accuracy for the outcome prediction. Then we compared the present model with the other eight models in the training, internal validation, and external validation cohorts. The present model showed a higher discrimination ability and fitness performance than all other models, and the 1- to-3-year AUROC of the present model was all higher than the other models separately. We compared the clinical usefulness of each model with the decision curve analysis. As shown in Figure 6, the present model we developed provided a larger net benefit compared with other models in the training, internal validation, and external validation cohorts.

### 3.4. Performance of the Model in Stratifying Risk of Patients

**Figure 7:**
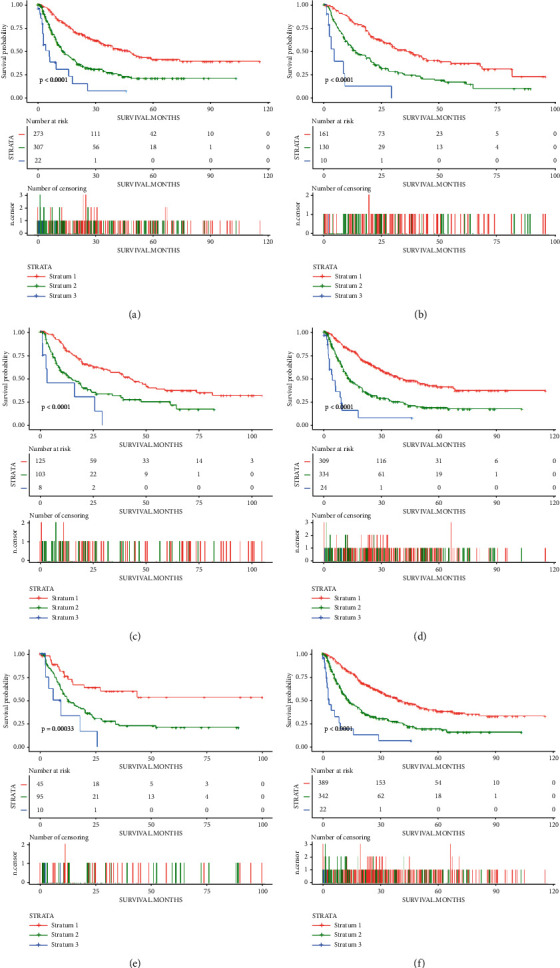
Kaplan–Meier curves of overall survival in patients from the subgroup of (a) ＜60 years old, (b) ≥60 years old, (c) AST level ≤ 40 U/L, (d) AST level ＞ 40 U/L, (e) Child–Pugh A, and (f) Child–Pugh B stratified by the risk strata; AST, aspartate aminotransferase.

We assigned a corresponding score to each selected prognostic factor of the model, based on its value. Then we calculated the total score for each individual according to the sum of the scores that were obtained from each risk factor. As shown in Table 4, patients were divided into three risk strata based on the score. The survival curve in Figure 7 revealed that patients in Stratum 1 had a better overall survival than Stratum 2, and the overall survival time of patients from Stratum 2 was better than that of Stratum 3 (*p* < 0.001). Then we compared the prognosis of patients from different strata in different subgroups based on age, the AST level, and the Child–Pugh class. As shown in Figure 7, the performance of the model in risk stratifying was still good in the subgroups.

## 4. Discussions

Patients with BCLC stage B HCC had a varied survival, hence several risk models or systems have been developed for the prediction of outcomes for those patients. The ALBI grade, as a surrogate of the CP grade, was evaluated to be a simple tool for the assessment of the liver function [9, 10]. However, it appears to be arbitrary and crude that the ascites variable which is contained in the CP grade, was eliminated from the ALBI grade. The present study revealed the prognostic value of ascites and combined that with the ALBI grade to get a new variable, the ALBI-AS grade. The ALBI-AS grade provided a well discriminatory ability. The three-year overall survival rate for patients of ALBI-AS grade C was 9.1% which was far below that of the ALBI grade II patients. Subsequently, the ALBI-AS grade along with the AFP level and the 8-and-14 grade were used for the development of a prognostic prediction model for patients with BCLC stage B HCC. The discrimination and fitness performance were investigated in the training cohort and verified in the internal and external validation cohorts, and then compared with the other eight models. The present ALBI-AS grade-based model provided an accurate prognostication and performed well against other prognostic models.

The liver function is a key parameter that would have influence on the survival of the patients with BCLC stage B HCC. The BCLC-B subclassification system and the new BCLC-B subclassification system adopted Child–Pugh score or class as the surrogate of the liver function [7, 8]. The ascites could be incorporated into the predictive model as a part of the CP score or class. And the patients of Child–Pugh B had a dismal prognosis compared with the patients of Child–Pugh A, owing to the high percentage of patients with ascites and clinical jaundice [25]. Recently, as the ALBI grade was proposed and incorporated into several HCC-related prognostic models, the ALBI grade has been regarded as a simple and pragmatic tool for assessing liver function rather than the CP grade. Therefore, the ascites variable was abandoned for not being part of the ALBI grade. And the prognostic value of the ascites was seldom evaluated in the studies on the ALBI related prognostic model, and there were even no studies on the survival analysis for the ascites when the ALBI grade was applied in the populations with BCLC stage B HCC. One of the reasons for this was that the most published studies on BCLC stage B or TACE either exclude or have limited inclusion of patients with decompensated cirrhosis, usually corresponding to the presence of ascites, and definitive conclusions regarding these patients cannot be made from the literature [25].

In fact, in Western countries, 90% of liver cancer occur in the background of cirrhosis, which itself is a progressive disease that affects the survival of patients [25]. The most serious complication of cirrhosis is portal hypertension. Ascites are the most common first symptom of liver decompensation, which seriously affects the prognosis of patients with cirrhosis [12]. Ascites were evaluated to be an independent risk factor for the survival of HCC patients, and have been incorporated into several HCC-related risk models [12, 13, 15, 17]. Therefore, there might be an over-simplification in the ALBI grade, and the ascites could be retained as the parameters for the assessment of the liver function. In the present study, both the ALBI grade and the ascites were used for the assessment of the liver function, and the predictive value of the ALBI-AS grade was acceptable. According to the model's performance comparison results, we could find out that the present ALBI-AS based model was a reasonable simplification of the Child–Pugh based models, and an improvement compared with the ALBI-based models.

We included patients with decompensated cirrhosis to develop a comprehensive prognosis model for patients with BCLC stage B HCC. TACE has been established as the standard of care for patients with BCLC stage B and was applied as the first-line treatment in our study [26, 27]. Decompensated cirrhosis was not considered to be an absolute contraindication to TACE. The study of Kim et al. revealed that decompensated patients with Child–Pugh class B (Child score 8 or 9) can benefit from TACE treatment if they have a limited tumor burden [8]. The HCC complicated with refractory ascites was believed to be a contraindication for the treatment of the TACE [28]. About 60% of cirrhotic patients develop ascites within 10 years, only ten percent of patients have refractory ascites. And there was a low percent of patients with refractory ascites, which part of patients were not included in the present study, in the BCLC stage B populations. TACE can be used for patients with marginal hepatic reserve (i.e., hyperbilirubinemia, ascites) [29]. Our study included patients with a small amount of ascites and no encephalopathy, which could be deemed as a marginal hepatic reserve. Hence, there was no heterogeneity in terms of primary treatment due to the inclusion of patients with decompensated cirrhosis. The TACE was not recommended for patients with HCC and ascites due to a more vulnerable chance of the ischemic injury after TACE [30]. However, the ascites variable was only one of the parameters that would have had an influence on survival. Patients of ALBI-AS grade B had a better prognosis than patients of ALBI-AS grade C, and patients of ALBI-AS grade C could also get a treatment benefit if they had a low AFP level or a better 8-and-14 grade. Through the prediction of the whole model, the prognosis of patients could be better evaluated, and patients suitable for TACE treatment could be screened out.

There were several limitations in the present study. First, the inherent limitations of the retrospective study; second, although the study was validated with multicenter data, all participants were from the Asian centers. Our findings should be further validated in the Western populations. Third, despite the included patients receiving the TACE as their first-line treatment, the additional treatments, such as radioembolization, targeted therapy, or ablation therapy, during the follow-up period could have had an influence on survival but not be controlled; fourth, the radiological ascites variable was used for the development of the present model. However, different radiological techniques (computed tomography or ultrasonography) and observers might have an influence on the results; fifth, the conventional regression methods were utilized in the present model, and the machine learning methods, which were believed to be flexible prediction algorithms, may be more accurate than the conventional regression and could be applied in the future studies on BCLC stage B HCC.

## 5. Conclusion

In summary, the ALBI-AS grade, as a pragmatic alternative of the ALBI grade, is a novel predictor of survival for patients with BCLC stage B HCC. The ALBI-AS based model was evaluated to be a useful prognostic tool for individual prognostication and performed well in terms of discrimination and fitness against other prognostic models. The present model could be applied to identify patients with BCLC stage B HCC that need aggressive treatment. However, it is appropriate to validate our findings in a larger prospective cohort.

## Figures and Tables

**Table 1:** The baseline characteristics of the BCLC stage B HCC patients from the training cohort.

The variables	The patients (*n* = 903)
Gender, *n* (%)	
Male	821 (90.9%)
Female	82 (9.1%)
Age (years), mean (SD)	53.2 (12.3)
HBsAg, *n* (%)	
Negative	111 (12.3%)
Positive	792 (87.7%)
Anti-HCV, *n* (%)	
Negative	885 (98.0%)
Positive	82 (9.1%)
HGB (g/L), mean (SD)	132.5 (19.7)
WBC (10^9^/L), median (IQR)	6.9 (5.2–9.3)
PLT (10^9^/L), median (IQR)	144.1 (99.0–202.0)
AST (U/L), median (IQR)	65.8 (39.6–125.5)
ALB (g/L), mean (SD)	38.7 (5.7)
TBLT(*μ*mol/L), median (IQR)	18.3 (12.6–27.1)
CRP (mg/L), median (IQR)	16.5 (3.4–55.4)
PT (seconds), mean (SD)	12.3 (1.3)
AFP (ng/ml), median (IQR)	242.3 (17.0–4447.5)
Size of main tumor (mm), median (IQR)	65.0 (43.0–95.5)
Number of lesions, *n* (%)	
≤3	360 (39.9%)
>3	543 (60.1%)
Ascites, *n* (%)	
No	867 (96.0%)
Little amount	33 (3.6%)
Middle amount	3 (0.4%)
Child–Pugh grade, *n* (%)	
A	150 (16.6%)
B	753 (83.4%)
ChildPugh score, *n* (%)	
≤6	150 (16.6%)
7	629 (69.7%)
8	89 (9.9%)
≥9	35 (3.9%)
ALBI score, mean (SD)	−2.4 (0.5)
APRI score, median (IQR)	1.5 (0.7–2.1)

HCC, hepatocellular carcinoma; HCV, hepatitis C virus; HGB, hemoglobin; WBC, white blood cell; PLT, platelet; AST, aspartate aminotransferase; ALB, albumin; TBLT, total bilirubin; CRP, C-reactive protein; PT, prothrombin time; AFP, alpha-fetoprotein; ALBI, albumin-bilirubin grade; APRI, AST to platelet ratio index; SD, standard deviation; IQR, interquartile range.

**Table 2:** The ALBI grade and the ALBI-AS grade.

The ALBI grade	Ascites	The ALBI-AS grade
I	No	A
	Yes	B

II	No	B
	Yes	C

**Table 3:** The comparison of the ALBI-AS based model versus other models for BCLC stage B HCC patients.

Model	1-yr AUROC	2-yr AUROC	3-yr AUROC	C-index (95% CI)	AIC
*Training group*					
The present model	0.73	0.69	0.67	0.68 (0.66–0.70)	6216.3
Up-to-seven	0.62	0.63	0.62	0.59 (0.57–0.61)	6290.2
Four-and-seven	0.65	0.63	0.62	0.62 (0.60–0.64)	6298.8
Six-and-twelve	0.66	0.64	0.62	0.63 (0.61–0.65)	5449.1
BCLC-B substaging system	0.60	0.59	0.59	0.59 (0.57–0.61)	6308.9
New BCLC-B substaging system	0.61	0.60	0.59	0.59 (0.57–0.62)	6320.8
HAP	0.60	0.59	0.58	0.58 (0.56–0.61)	6351.1
mHAP II	0.56	0.56	0.55	0.55 (0.53–0.57)	6357.3
ALBI-TAE model	0.67	0.65	0.64	0.64 (0.62–0.67)	6283.5

*Internal validation group*					
The present model	0.72	0.71	0.69	0.70 (0.67–0.74)	2306.2
Up-to-seven	0.62	0.61	0.60	0.61 (0.59–0.63)	2335.4
Four-and-seven	0.65	0.63	0.61	0.63 (0.59–0.66)	2356.8
Six-and-twelve	0.64	0.64	0.63	0.63 (0.60–0.66)	2339.3
BCLC-B substaging system	0.64	0.63	0.62	0.63 (0.59–0.66)	2336.1
New BCLC-B substaging system	0.65	0.63	0.62	0.64 (0.60–0.67)	2346.5
HAP	0.61	0.61	0.59	0.60 (0.56–0.63)	2376.8
mHAP II	0.57	0.58	0.56	0.57 (0.54–0.60)	2381.4
ALBI-TAE model	0.69	0.67	0.65	0.67 (0.63–0.70)	2334.7

*External validation group*					
The present model	0.71	0.70	0.68	0.67 (0.64–0.71)	2056.6
Up-to-seven	0.61	0.60	0.59	0.59 (0.56–0.61)	2090.2
Four-and-seven	0.68	0.66	0.65	0.65 (0.62–0.68)	2074.3
Six-and-twelve	0.67	0.65	0.63	0.63 (0.60–0.66)	2082.8
BCLC-B substaging system	0.63	0.61	0.60	0.61 (0.57–0.63)	2091.1
New BCLC-B substaging system	0.66	0.64	0.63	0.63 (0.59–0.66)	2078.7
HAP	0.60	0.59	0.59	0.59 (0.54–0.62)	2107.3
mHAP II	0.57	0.57	0.56	0.56 (0.23–0.59)	2106.7
ALBI-TAE model	0.67	0.66	0.65	0.64 (0.60–0.68)	2081.9

AUROC, area under the receiver operating characteristics curves; CI, confidence interval; AIC, akaike information criterion.

**Table 4:** The risk stratification by the ALBI-AS based model in the BCLC-B HCC patients.

Score	0	1	2

AFP (ng/mL)	<400	≥400	
ALBI-AS grade	A	B	C
8-and-14 grade	A	B	C

The total score	Strata		

0-1	Stratum 1		
2-3	Stratum 2		
4-5	Stratum 3		

## Data Availability

Data are available from the corresponding author upon request.

## Abbreviations

DCA: Decision curve analysis
HR: Hazard ratio (HR)
AUROC: Area under the receiver operator characteristic curve
C-index: Concordance index
AIC: Aiken information criterion
HCC: Hepatocellular carcinoma
BCLC: Barcelona clinic liver cancer staging system
CP: Child–Pugh grade
ALBI: Albumin-bilirubin grade
APRI: AST to platelet ratio index
IQR: Interquartile range.

## Supplementary Materials

Supplementary Materials Supplementary Table 1: the baseline characteristics of the BCLC stage B HCC patients from the internal and external validation cohort. HCC, hepatocellular carcinoma; AST, aspartate aminotransferase; ALB, albumin; TBLT, total bilirubin; PT, prothrombin time; AFP, alpha-fetoprotein; ALBI, albumin-bilirubin grade; SD, standard deviation; IQR, interquartile range. Supplementary Figure 1: the X-tile analysis for the identification of the optimal cutoff values of the ALBI score in the A) internal cohorts and B) external cohorts. Supplementary Figure 2: the X-tile analysis for the identification of the optimal cutoff values of the sum of the size of the largest tumor and the number of tumors in the A) internal cohorts and B) external cohorts. Supplementary Figure 3: Kaplan–Meier curves of overall survival in patients with BCLC stage B HCC stratified by A) the AFP level, B) the ALBI-AS grade, and C) the 8-and-14 grade.
