# Supplementary material for: An ALBI- and Ascites-Based Model to Predict Survival for BCLC Stage B Hepatocellular Carcinoma
Source: Evid Based Complement Alternat Med. 2022 Jul 7;2022:1801230. doi: 10.1155/2022/1801230 (PMC9283054; doi:10.1155/2022/1801230)

**Supplementary Table 1.** The baseline characteristics of the BCLC stage B HCC patients from the internal and external validation cohort.

| **The Variables** | **The Internal Validation Cohort (n=527)** | **The external Validation Cohort (n=343)** |
| --- | --- | --- |
| **Gender, n (%)** |  |  |
| **Male** | 131 (24.9%) | 286 (83.4%) |
| **Female** | 396 (75.1%) | 57 (16.6%) |
| **Age (years), mean (SD)** | 53.2 (11.6) | 52.0 (11.9) |
| **AST (U/L), median (IQR)** | 61.1 (36.2-113.5) | 63.0 (38.0-119.0) |
| **ALB (g/L), mean (SD)** | 38.7 (5.0) | 38.7 (6.0) |
| **TBLT (umol/L), median (IQR)** | 16.7 (11.9-25.5) | 19.4 (12.9-27.2) |
| **PT (seconds), mean (SD)** | 12.2 (1.2) | 12.3 (1.2) |
| **AFP (ng/ml), median (IQR)** | 253.9 (19.8-5038.0) | 210.2 (12.0-1988.0) |
| **Size of main tumor (mm), median (IQR)** | 63.0 (41.0-92.5) | 67.0 (43.0-96.5) |
| **Number of lesions, n (%)** |  |  |
| **≤3** | 201 (38.1%) | 124 (36.2%) |
| **>3** | 326 (61.9%) | 219 (63.8%) |
| **Ascites, n (%)** |  |  |
| **No** | 508 (96.4%) | 326 (95.0%) |
| **Yes** | 19 (3.6%) | 17 (5.0%) |
| **Child-Pugh grade, n (%)** |  |  |
| **A** | 468 (88.8%) | 298 (86.9%) |
| **B** | 59 (11.2%) | 45 (13.1%) |
| **Child-Pugh score, n (%)** |  |  |
| **≤6** | 468 (88.8%) | 298 (86.9%) |
| **7** | 36 (6.8%) | 29 (8.5%) |
| **8** | 19 (3.6%) | 16 (4.7%) |
| **≥9** | 4 (0.8%) | 0(0%) |
| **ALBI score, mean (SD)** | -2.5 (0.5) | -2.4 (0.6) |

HCC, hepatocellular carcinoma; AST, aspartate aminotransferase; ALB, albumin; TBLT, total bilirubin; PT, prothrombin time ; AFP, alpha-fetoprotein; ALBI, albumin-bilirubin grade; SD, standard deviation; IQR, interquartile range.

**Supplementary Figure 1.** The X-tile analysis for the identification of the optimal cutoff values of the ALBI score in the A) internal cohorts and B) external cohorts.


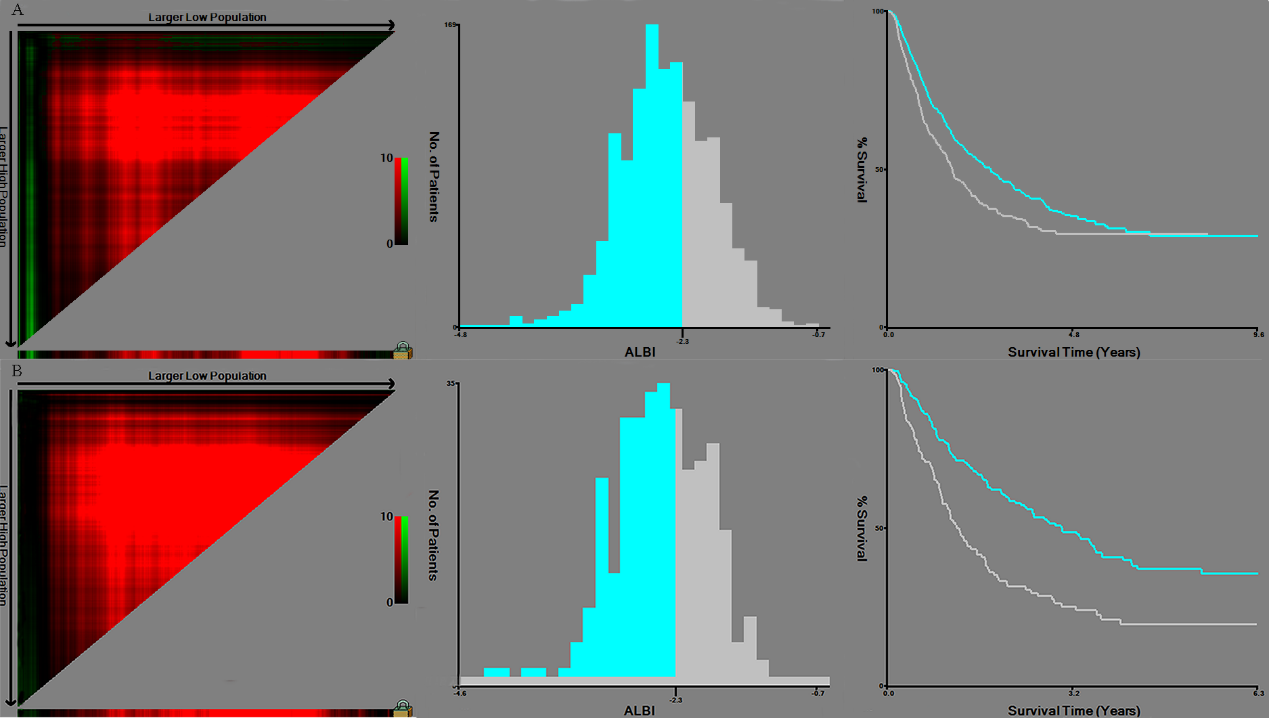


**Supplementary Figure 2.** The X-tile analysis for the identification of the optimal cutoff values of the sum of the size of the largest tumor and the number of tumors in the A) internal cohorts and B) external cohorts.


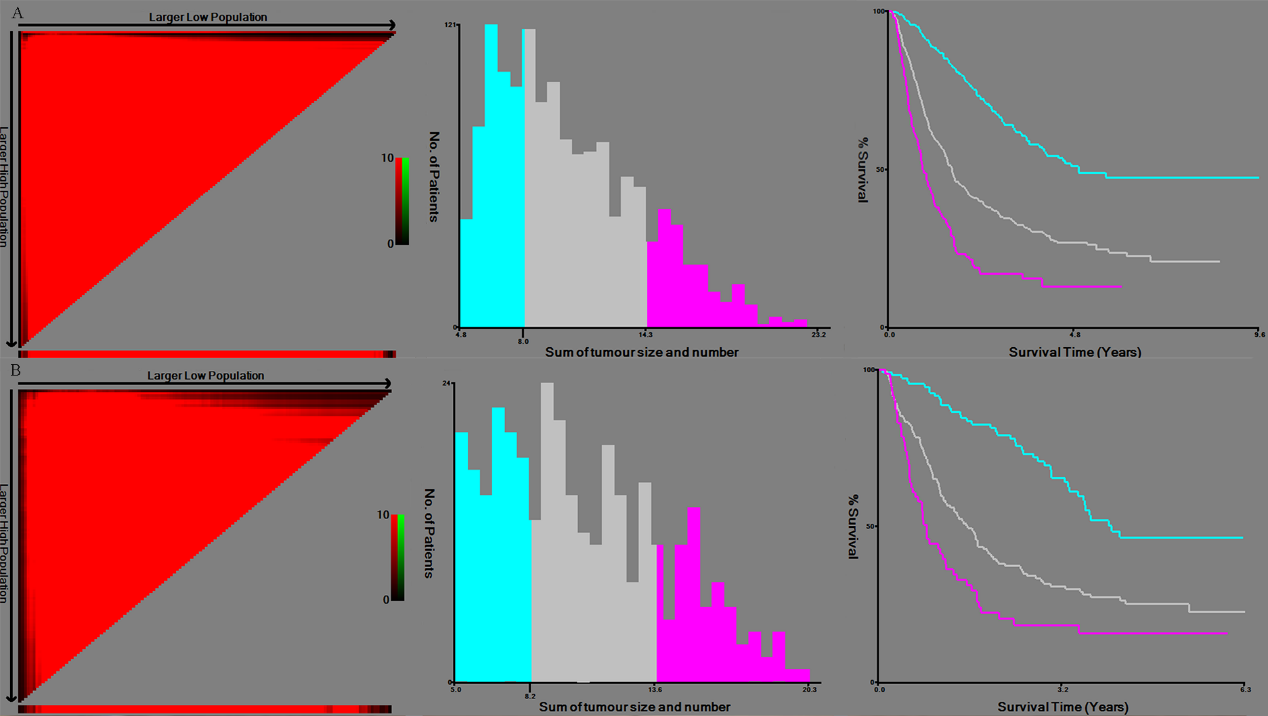


**Supplementary Figure 3.** Kaplan-Meier curves of overall survival in patients with BCLC stage B HCC stratified by A) the AFP level, B) the ALBI-AS grade and C) the 8-and-14 grade.


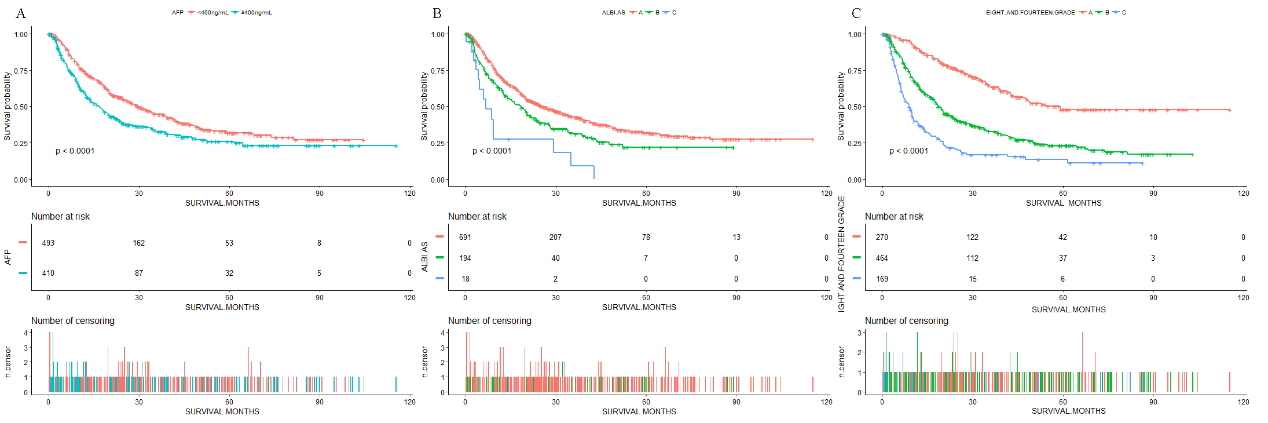

Supplement: Supplementary Materials — Supplementary Table 1: the baseline characteristics of the BCLC stage B HCC patients from the internal and external validation cohort. HCC, hepatocellular carcinoma; AST, aspartate aminotransferase; ALB, albumin; TBLT, total bilirubin; PT, prothrombin time; AFP, alpha-fetoprotein; ALBI, albumin-bilirubin grade; SD, standard deviation; IQR, interquartile range. Supplementary Figure 1: the X-tile analysis for the identification of the optimal cutoff values of the ALBI score in the A) internal cohorts and B) external cohorts. Supplementary Figure 2: the X-tile analysis for the identification of the optimal cutoff values of the sum of the size of the largest tumor and the number of tumors in the A) internal cohorts and B) external cohorts. Supplementary Figure 3: Kaplan–Meier curves of overall survival in patients with BCLC stage B HCC stratified by A) the AFP level, B) the ALBI-AS grade, and C) the 8-and-14 grade. [file 1801230.f1.docx]
